# Using supply side evidence to inform oral artemisinin monotherapy replacement in Myanmar: a case study

**DOI:** 10.1186/s12936-016-1385-4

**Published:** 2016-08-18

**Authors:** Hnin Su Su Khin, Tin Aung, Moe Aung, Aung Thi, Matt Boxshall, ACTwatch Group, Chris White

**Affiliations:** 1 Population Services International Myanmar, No. 16, West Shwe Gon Taing Street 4, Yangon, Myanmar; 2 National Malaria Control Programme, Department of Public Health, Ministry of Health and Sports, Naypyidaw, Myanmar; 3 Marie Stopes International, 1 Conway Street, Fitzroy Square, London, W1T 6LP UK; 4 Population Services International, 1120 19th St NW Suite 600, Washington, DC 20036 USA; 5 Division of Global Policy and Advocacy, Bill & Melinda Gates Foundation, Seattle, WA USA

**Keywords:** Myanmar, Antimalarial drug resistance, Malaria elimination, Program design, Supply survey, Outlet survey

## Abstract

**Background:**

In 2012, alarmingly high rates of oral artemisinin monotherapy availability and use were detected along Eastern Myanmar, threatening efforts to halt the spread of artemisinin resistance in the Greater Mekong Subregion (GMS), and globally. The aim of this paper is to exemplify how the use of supply side evidence generated through the ACTwatch project shaped the artemisinin monotherapy replacement malaria (AMTR) project’s design and interventions to rapidly displace oral artemisinin monotherapy with subsidized, quality-assured ACT in the private sector.

**Methods:**

The AMTR project was implemented as part of the Myanmar artemisinin resistance containment (MARC) framework along Eastern Myanmar. Guided by outlet survey and supply chain evidence, the project implemented a high-level subsidy, including negotiations with a main anti-malarial distributor, with the aim of squeezing oral artemisinin monotherapy out of the market through price competition and increased availability of quality-assured artemisinin-based combinations. This was complemented with a plethora of demand-creation activities targeting anti-malarial providers and consumers. Priority outlet types responsible for the distribution of oral artemisinin monotherapy were identified by the outlet survey, and this evidence was used to target the AMTR project’s supporting interventions.

**Conclusions:**

The widespread availability and use of oral artemisinin monotherapy in Myanmar has been a serious threat to malaria control and elimination in the country and across the region. Practical anti-malarial market evidence was rapidly generated and used to inform private sector approaches to address these threats. The program design approach outlined in this paper is illustrative of the type of evidence generation and use that will be required to ensure effective containment of artemisinin drug resistance and progress toward regional and global malaria elimination goals.

## Background

The malaria burden in Myanmar is far higher than in any other country in South East Asia, accounting for most of the reported malaria cases in the Greater Mekong Sub-region (GMS) [1]. Malaria has been the leading cause of morbidity and mortality in Myanmar for over two decades [2]. Around 35 million people, or 70 % of the population, are at risk of malaria, and 25 million are considered at high risk. Malaria risk is higher in forested and hilly areas such as the areas in Eastern Myanmar, areas mainly inhabited by ethnic minorities [3]. The most vulnerable are migrant workers, particularly those who work in and around forests, in industries such as gem-mining, logging, agriculture and construction.

Artemisinin-based combination therapy (ACT) has helped to reduce the global burden of malaria, however this is now threatened by the emergence of malaria parasites that are resistant to artemisinin [4, 5]. Of grave concern is that artemisinin-resistant *Plasmodium falciparum* has been identified on the eastern borders of Myanmar [6] and, most recently, along the border with India [7]. Myanmar is thus critical to global efforts to contain resistance, but the complexities are vast. There is extensive migration in high transmission areas, increasing the spread of resistance. The country has been faced with over 60 years of civil conflict in some border areas. Inadequate investment in the health system—overall and for malaria control in particular—has posed challenges to effective malaria case management [8].

In 2011, there was little information on the private sector in Myanmar, but it was estimated that most malaria treatments in Myanmar were accessed directly from this sector, similar to other markets in the region [9]. Low levels of government investment and conflict, particularly along the remote border areas where malaria is highest, meant that health services were of limited quality or availability. Thus, it was anticipated that local drug sellers and other private sector provider types were typically sought—after for treatment [8]. There was also concern that most malaria was not only self-diagnosed but treated inappropriately with incomplete courses of oral artemisinin monotherapy. The use of oral artemisinin monotherapy, incomplete courses  and, in particular, substandard drugs was of concern given emerging drug resistance.

To address this situation, in 2010, the Government of Myanmar, under the Myanmar artemisinin resistance containment (MARC) framework, developed a comprehensive set of interventions to address the spread of artemisinin resistance in Eastern Myanmar [3]. As part of the private sector initiative, in 2012, Population Services International (PSI), a large, US-based NGO, designed and implemented the evidence-based artemisinin monotherapy replacement (AMTR) malaria project with support from the UK Department For International Development (DFID), the Bill and Melinda Gates Foundation (BMGF) and Good Ventures. At a global level, the AMTR project aimed to ensure a firewall against the spread of artemisinin resistance through South East Asia, India and to Africa; and at a national level, it aimed to increase the availability and supply of high quality, affordable ACT, thus reducing the burden of *P. falciparum* in the endemic areas and moving the country from control towards elimination of malaria. In its simplest form, the AMTR project approach was to get subsidized, high-quality-assured, first-line ACT into the private sector through key suppliers and distributors, with the aim of squeezing oral artemisinin monotherapy out of the market due to price competition, increased availability of ACT, and other demand-creation activities.

This paper documents how two critical research studies informed the development and design of the AMTR project across Eastern Myanmar and describes the program activities that were developed as a result of the malaria landscape in 2010–2012, using evidence that these research studies generated.

### The studies

Prior to the program implementation, two supply side surveys, a supply chain study and an outlet survey were conducted in Eastern Myanmar to investigate the private sector anti-malarial landscape, with the aim of using the findings to inform programming decisions and implementation activities. The studies are described in this sub-section.

#### The supply chain study

In 2010, PSI/Myanmar conducted a supply chain study to document the distribution chain for anti-malarials in Eastern Myanmar. The aim of the supply chain study was to provide a snapshot of the current market for anti-malarial drugs in Eastern Myanmar: how the supply chain functioned, how the anti-malarial medicines were bought and sold, which anti-malarials were dominating the market, and estimated prices to patients as well as mark-ups.

The supply chain study was conducted primarily to provide input into the proposal development for the project and was used as formative research to help shape the project development and rationale. A short, open-ended interview guide was developed to ascertain information from key informants across different levels of the supply chain. Questions included: where the anti-malarials medicines were purchased from, the quantity/amount purchased, the quantity/amount typically distributed, and the type of anti-malarial medicine. Providers who sold to patients were asked the typical selling price. Snowball and purposive sampling methods were used. The assessment utilized a bottom-up approach to identify respondents. Initially, respondents (key informants) at the bottom end of the supply chain were interviewed, namely general retailers and mobile providers. This was the first wave of data collection. A second wave of interviews was implemented with pharmacists and wholesalers that had been identified in the first wave of data collection by the general retailers and mobile providers. Finally, a third wave of data collection was implemented with the higher-order wholesalers and distributors referred to by the key informants in the second wave of interviews. The assessment allowed for a rapid assessment of the supply chain. In total, 34 key informants were interviewed: 11 mobile providers, 8 general retailers, 9 pharmacies, 3 wholesalers and 3 importers and distributors. Data were analyzed and transcribed using standard qualitative thematic methods but focused specifically on identifying the main distributor(s) and a diagrammatic qualitative representation of the supply chain structure.

**Fig. 1:**
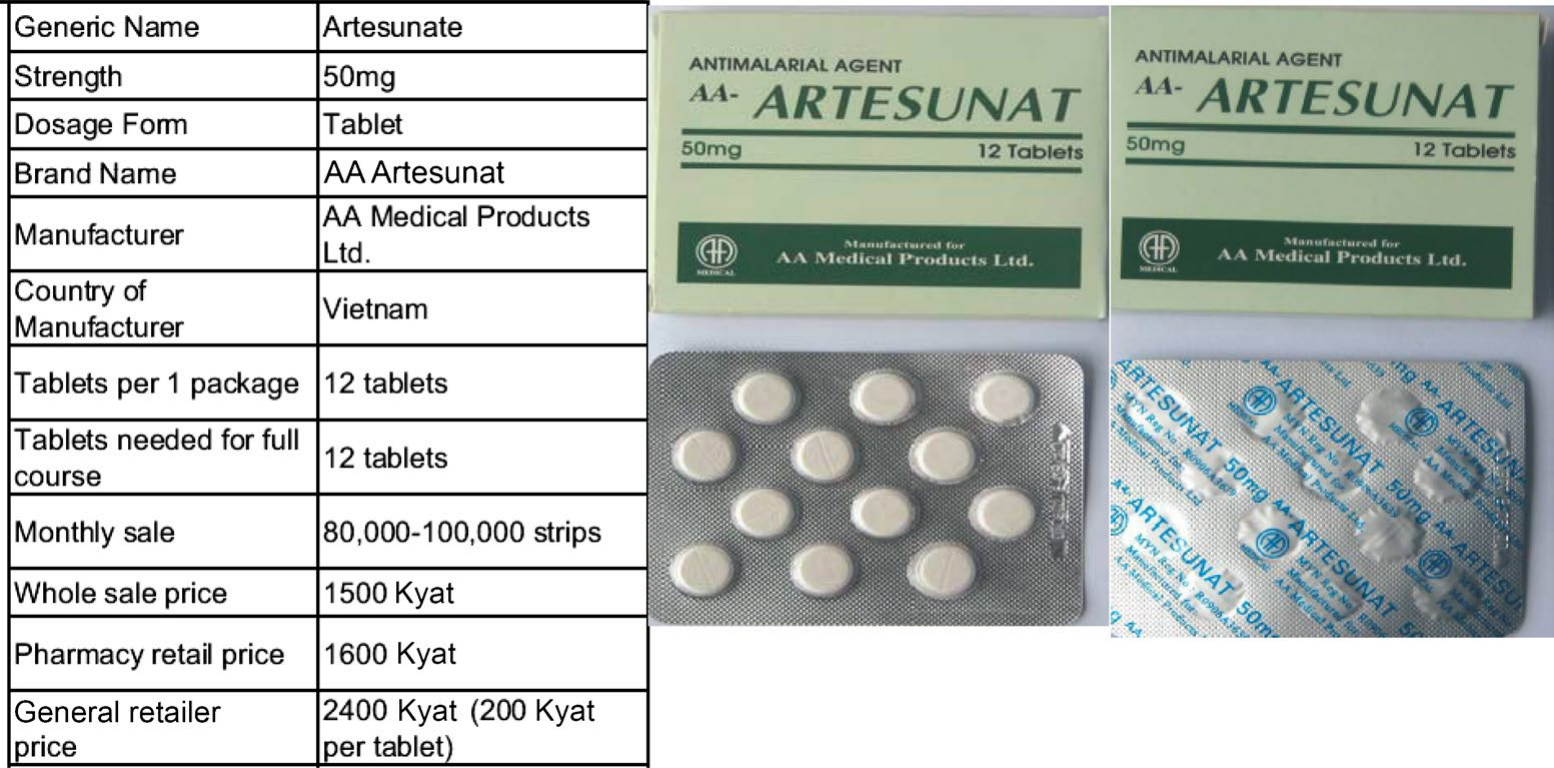
Samples of common artesunate monotherapy identified by the supply chain

The first study took place along the eastern border, the area with the highest risk of drug resistance. A second study was conducted close to the China border, in Kachin and Shan States, to investigate whether medicines from China played a significant part in the local market. Both studies confirmed a very similar pattern. A core finding was that 70 % of the artemisinin monotherapy in the anti-malarial market was supplied by one distributor: AA Medical Products Limited. The main product being supplied by this wholesaler was called ‘AA Artesunat^®^, (Fig. 1), although other artemisinin monotherapy products were identified such as Artem^®^ (Artemether injection and tablets), Arthesis^®^, AA-Artemether^®^ and Artemodi^®^ (all oral artemisinin tablets). This distributor not only had control of the artemisinin monotherapy market but also had a sophisticated distribution mechanism that penetrated outlets that were directly serving patients, such as general retailers and pharmacies. Many providers in towns and cities in the area were supplied directly by the AA Medical Products Limited distribution mechanism. It was also observed that half of the sales were funneled through wholesalers, and the artemisinin monotherapy distribution was therefore relatively centralized.

Regarding information on sales from the supplier, almost all of the anti-malarials distributed by AA Medical Products Limited were oral artemisinin monotherapy. It was estimated that more than 100,000 strips (of 12 tablets) of artesunate monotherapy were distributed per month on average—and around 1.2 million doses were imported per annum.

Other evidence gathered across the supply chain helped to set the eventual price of the subsidized, quality-assured ACT. Data showed that the average mark-up of anti-malarial drugs across the different levels of the supply chain varied. Among outlets serving patients, it was feasible to determine how many oral artemisinin tablets were typically sold to patients and at what price. It was estimated that the market price of a full adult course of artesunate, comprised of 12 tablets, was around 2400 (Myanmar) Kyat. However, providers reported that they typically only gave two or three tablets to the patient, preferring to sell individual pills rather than a complete dose (with two to three pills sold for around 500 Kyat or $0.40 in 2012). Interviews with providers suggested that each strip of artesunate monotherapy was typically used to treat two to three malaria-suspected patients. This evidence on price of sale to patients, as well as mark-ups along the distribution chain, helped the program to calculate in a backward fashion a recommended retail price for the quality-assured ACT.

Other evidence from key informant interviews conducted with patients indicated that the private sector played a major role in malaria treatment in Myanmar. Most commonly, patients described purchasing anti-malarial medicines from local private sector outlets and were unlikely to receive a malaria diagnostic test. If high-quality ACT treatment drugs were available at these outlets, they were described as prohibitively expensive. Providers also reported that they gave clients ‘what they wanted’, and this was typically artemisinin in tablet form. The root cause of the sale of incomplete treatment regimens was most often described as ‘cost’, as patients could not afford a full course.

#### The outlet survey

A cross-sectional outlet survey based on the ACTwatch methodology was conducted across 26 townships in Eastern Myanmar between March and May, 2012 [10]. The objective of the ACTwatch outlet survey was to investigate the availability, price and market share of anti-malarial medicines across private sector outlets. The methods and study objectives have been described in detail elsewhere [11–13]. Briefly, a census of all outlets with the potential to sell anti-malarials was conducted in selected townships and wards across Eastern Myanmar. Among eligible outlets, that is, outlets that stocked anti-malarial medicines on the day of survey or in the previous 3 months, a full audit of anti-malarials was conducted. Information on the brand and generic name, strength, amount sold in the previous week as well as the price was collected for each anti-malarial medicine. A provider interview assessing provider knowledge and dispensing practices was also implemented.

**Fig. 2:**
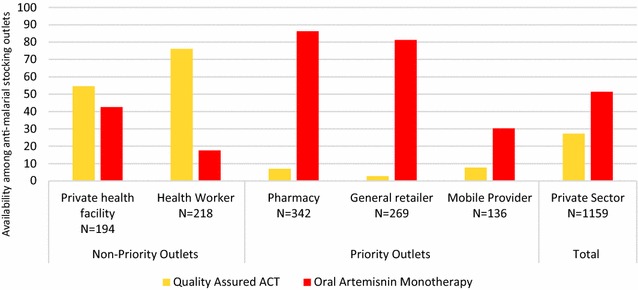
Availability of different classes of anti-malarials, Eastern Myanmar 2012

A total of 3658 private sector outlets with the potential to sell anti-malarials were screened. In the context of Myanmar, these included private facilities and community health workers, pharmacies, general retailers and mobile providers (or sometimes described as itinerant drug vendors who do not typically operate from a fixed location and may or may not have some medical background/training). Of the 3658 outlets screened, 32 % stocked at least one anti-malarial at the time of the study, including 82 % of private health facilities (N = 273), 73 % of community health workers (N = 348), 79 % of pharmacies (N = 454), 55 % of mobile providers (N = 290), and 15 % of general retailers (N = 2292). Availability was relatively low among general retailers (village stores and grocery stores) although they were the most numerous outlet type in the census. The availability of quality-assured ACT varied across different anti-malarial-stocking outlet types: private health facility (55 %), community health worker (76 %), pharmacy (7 %), mobile provider (8 %) and general retailer (3 %). Availability of oral artemisinin monotherapy was higher across all key outlet types: pharmacy (86 %), mobile provider (30 %), general retailer (81 %) (see Fig. 2). Priority outlets—pharmacies, general retailers, and mobile providers—were outlets that received additional supportive interventions, namely production promotion through PSI.

**Fig. 3:**
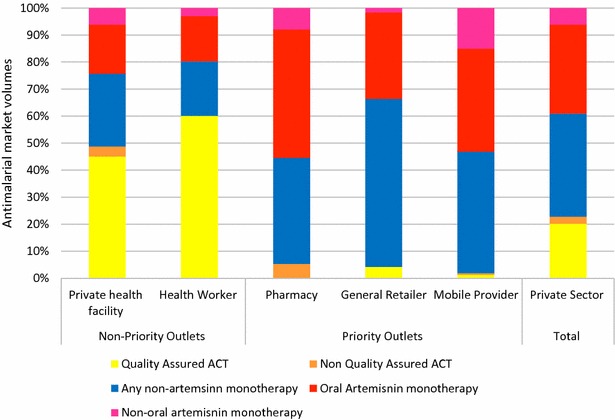
Market share of anti-malarials distributed in the past week, Eastern Myanmar 2012

Observing market share of different classes of anti-malarials, oral artemisinin monotherapy accounted for 33 % of the total market share. Within outlets, oral artemisinin was most commonly sold by pharmacies (more than 45 % of the market share), mobile providers (38 % of the market share), and general retailers (32 % of the market share) (Fig. 3). Across these three outlet types, market share of quality-assured ACT was less than 5 % among mobile providers and general retailers, and was reportedly not sold or distributed in the previous week by pharmacies. Provider knowledge of the first-line treatment for malaria was exceptionally low among anti-malarial-stocking pharmacies, mobile providers and general retailers (<10 %) compared to private facilities and community health workers (58.2 and 54.9 %, respectively). Another finding was that providers often report cutting blisters of pills, lending to cocktail formulations or other sub-optimal treatment regimens, and this was most common in pharmacies, general retailers and among mobile providers [10].

In summary, the supply side data indicate that there were a number of barriers to ensuring access to the first-line ACT treatment for malaria, including availability and price of ACT. The supply side data indicated wide spread use and distribution of other anti-malarials to treat malaria, in particular oral artemisinin monotherapy, which is acknowledged as a major bottleneck in containing resistance to artemisinin derivatives in Myanmar. The findings from the supply chain and the outlet survey were used to inform the AMTR project strategy for oral artemisinin monotherapy replacement in the private sector and are discussed in the next section.

### How evidence and policy shifts were used to develop and design the AMTR project activities

#### Enabling environment

In 2010, at the time of the supply chain rapid assessment, artemisinin monotherapy was legal and registered by the Government of Myanmar, although the national guidelines encouraged use of a combination therapy. Findings from the supply chain provided evidence regarding the distribution of oral artemisinin monotherapy in Eastern Myanmar. PSI, along with other organizations and partners within the MARC framework, shared the findings with the NMCP program managers as a means to help senior officials within the government advocate for tightening the importation and regulation of artemisinin monotherapy so that only intravenous, intramuscular and rectal suppository formulations of artemisinin for the treatment of severe malaria would be permitted. Subsequently, the importation of oral formulations of artesunate and artemether was banned in Myanmar in December 2011, and August 2012, respectively, by the Food and Drug Administration (FDA). However, oral artemisinin monotherapy was still legal to purchase in Myanmar while existing stocks were used up.

#### Theory of change

**Fig. 4:**
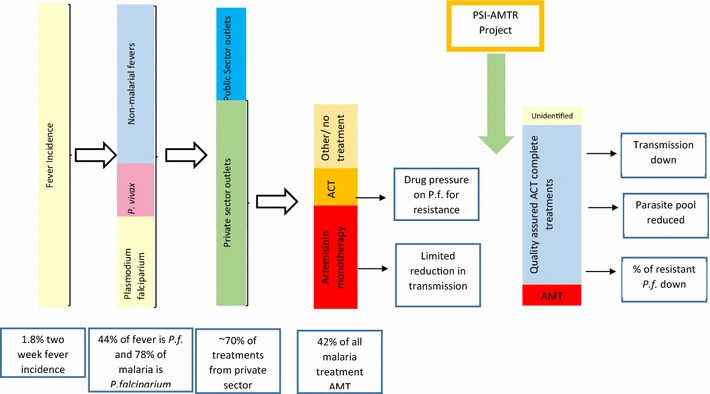
PSI Theory of change during the project inception

The AMTR project was also guided by a theory of change, pertaining to how the interventions and activities implemented by PSI would shape the market over time. While it is acknowledged at the time of writing that some of these assumptions have changed based on new epidemiological data, the framework helped to guide and visualize the project implementation and vision of success. The framework was developed using information from the supply chain as well as other key resources from the Ministry of Health and the World Health Organization. The following situation was described (see Fig. 4): (1) Based on the number of malaria cases visited in public health facilities and the number of annual oral artemisinin monotherapy drugs imported into the country, it was roughly estimated that there were 5 million fever cases in the PSI target area—equivalent to a two-week fever prevalence of 1.8 % among the general population; (2) Of these fever cases, 44 % test positive for *P. falciparum* and 78 % of all malaria cases are infections with *P. falciparum*; (3) The majority of all cases were treated in the private sector (estimated to be around 70 %); (4) Of malaria treatments from the private sector, the majority of treatments given were oral artemisinin monotherapy, and most patients only took a partial dose; (5) This low dose of oral artemisinin monotherapy was limited in its effects on malaria transmission and represented a tremendous drug pressure on the malaria parasites, enhancing the likelihood of selecting resistant strains, and; (6) Reversing this situation by replacing oral artemisinin monotherapy in the private sector with full treatment courses of quality-assured ACT through the AMTR project would then significantly contribute to the resistance-containment efforts.

#### Stopping oral artemisinin monotherapy distribution

Based on the findings of the supply chain survey, in 2012, PSI engaged the major private sector supplier of artemisinin, AA Medical Products Limited, in an agreement to purchase highly-subsidized, pre-packaged, quality-assured ACT from PSI. This was expected to rapidly replace oral artemisinin monotherapy in at least 70 % of all private sector malaria treatment providers in Eastern Myanmar, especially given the extensive distribution networks that were already in place.

#### Developing the right products

According to national treatment guidelines, the ACT artemether-lumefrantrine (AL) was indicated for the treatment of *P. falciparum* malaria. WHO prequalified AL tablets were procured through a competitive bid and over-branded with the brand names Supa Arte^®^ and Artel^®^. Both products were first-line treatment for uncomplicated malaria and packaged according to four child/adolescent and adult weight ranges. In addition to this, an unbranded generic ACT was also distributed in the private sector directly to PSI-franchised Sun Quality Health Clinics. Having two different brands helped to ensure that providers further down the supply chain had a choice between different malaria treatments. This facilitated the progressive replacement of oral artemisinin monotherapy by ACT in the private sector and also created healthy competition.

Both brands were marked with a quality seal—the Padonmar (lotus leaf), designed to identify all quality-assured ACT imported and distributed in Myanmar, allowing for the program to promote the anti-malarial medicine rather than a particular formulation, manufacturer, distributor or/and brand. The NMCP and the FDA were also closely involved in this process, as the seal was meant to be used by all partners distributing quality-assured ACT in the country—regardless of the donor funding or project location. Thus, any WHO-approved ACT that was part of the national treatment guidelines was branded and identified using the lotus leaf seal.

A key advantage of the quality seal was that it also enabled PSI and other partners to potentially respond to changing national guidelines of first-line ACT without eroding brand equity, should sentinel surveillance suggest that partner drug efficacy was deteriorating. It was also created to help both providers and consumers identify the best possible treatment for *P. falciparum* malaria. In addition, the 30 % of the market that was not being reached by the key drug distributors would still be able to know that they were accessing a quality, first-line ACT. The quality seal was developed with participation from over 14 stakeholder organizations, including the NMCP, and the content and design were field-tested with the target audience.

### How the quality-assured ACT were manufactured and distributed

**Fig. 5:**
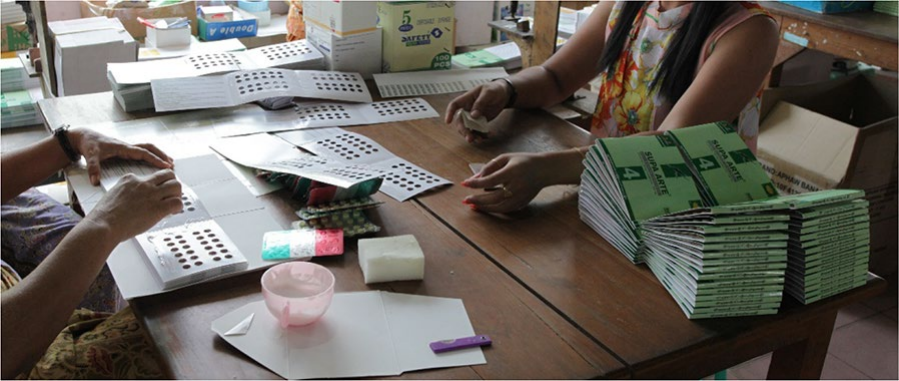
Over-packaging ACT treatments in PSI the warehouse

The quality-assured ACT were procured directly by PSI through a competitive bid. The imported ACT were over-packaged (additional packaging with consumer instructions) and over-branded in Myanmar in PSI’s own warehouse (Fig. 5). The over-packaging was done with the aim of deterring health care providers from cutting the blister packets and using ineffective single doses, as evidence from the supply surveys suggested this was a common practice.

After over-packaging, AA Medical Products Limited collected the quality-assured ACT branded products from the PSI warehouse and distributed them through their existing networks.

### Ensuring affordable anti-malarials

Both of the quality-assured ACT brands were highly subsidized so that patients were motivated to purchase them over other anti-malarial medicines. The subsidy was provided to the extent that the price of a complete dose of quality-assured ACT was equivalent to the same price of a partial dose (2–3 tablets) of oral artemisinin monotherapy, which was typically taken by febrile patients with suspected malaria. The recommended retail selling price was 500 Kyat, reflecting a subsidy of 80 % of the regular non-subsidized price. The price was determined based on the information from the supply chain survey, including average mark-ups across different levels and the number of tablets typically sold to consumers by lower-level suppliers. Acknowledging that people with suspected malaria can typically afford around 500 Kyat, the program calculated the price in a backward fashion, from the lower-level suppliers to the wholesale/distributor level, to determine a price that AA Medical Products Limited would use to sell to its wholesalers. The recommended retail price for quality-assured ACT was then monitored using the annual outlet surveys, reporting on the percentage of providers that sold a full course quality-assured ACT at or below 500 Kyat. Data from subsequent outlet surveys revealed that this assumption was correct, with the majority of priority outlets (~80 %) selling a full course of quality-assured ACT at or below 500 Kyat.

### Creating consumer demand

At a national level, mass media communication campaigns were implemented to encourage the public to demand quality ACT from drug sellers. These campaigns raised awareness of the need to seek prompt treatment and to take a full course of an effective and affordable ACT and highlighted the problems associated with consumption of counterfeit drugs. At the time of implementation, the majority of febrile patients had been using monotherapies for over a decade. The main purpose of the communication campaign was to create demand for a full course of pre-packaged quality-assured ACT rather than loose tablets (and incomplete doses) of a monotherapy. A key aim was to ensure that patients could easily identify a ‘quality-assured’ ACT from other products already in the market and being sold by providers, including other ACT that may not be quality-assured. Thus, a core component of the communication campaign was to create demand and awareness of the ‘lotus leaf’ quality seal. All communication and campaign messages were around WHO/NMCP-recommended quality-assured ACT, which could be recognized by the lotus leaf quality seal. To ensure messaging resonated with a variety of target audiences, the communication campaigns included a variety of people, such as migrant workers and rubber tappers, all of whom spoke to the benefits of the quality seal and of adherence to the full course.

### Targeting the key areas and promoting provider behavior change

The AMTR project was national in scope, as the supply chain intervention reached all states and regions with quality-assured ACT. However, in the eastern part of the country, including the MARC high-risk zones (zones identified as most at risk of artemisinin resistance in Myanmar), PSI designated a target area for an intensified communications campaign. The target area covered a population of a little over ten million people and included all of the MARC-defined high-risk zones and surrounding and linking townships.

In this target area, ACT sales and mass media were reinforced by pharmaceutical detailing operations through PSI product promoters. Product promoters implemented provider behavior change and interpersonal communication as a means to amplify an increase in ACT uptake in the supply chain. At the project inception, PSI recruited and trained over 75 product promoters to specifically target the different priority outlets [pharmacies, general retailers and mobile providers (see below for further discussion)] and to promote awareness and knowledge of quality-assured, effective ACT among providers from these outlets. The product promoters spoke local languages, which helped to ensure ethnic minority communities were reached. The product promoters provided information on general malaria transmission and prevention, malaria drug resistance, and stocking and dispensing decisions and also provided key messages around the need to use ACT and the harm associated with incorrect drug regimes. Product promoters also shared information regarding the risks associated with use of oral artemisinin monotherapy and the benefits of prescribing a full course of quality-assured ACT. They specifically discouraged the stocking of oral artemisinin monotherapy and promoted the replacement of existing stocks with quality-assured ACT. Product promoters were also responsible for provision of information, education and communication (IEC) materials pertaining to malaria treatment, as well as job aid materials. They also collected routine sales data of quality-assured ACT and oral artemisinin monotherapy.

Product promoters did not sell ACT directly to the priority outlets but did provide free samples of quality-assured ACT on their first visit and then established a link between the outlets and the supply points by providing providers with a list of the wholesalers and mid-wholesalers from the nearest towns or areas where quality-assured ACT could be purchased. They also provided providers with a recommended retail selling price. Product promoters visited all of the AMTR priority outlets in their assigned township every quarter.

As well as targeting the priority outlets, product promoters also visited the wholesalers in the surrounding towns to ensure the availability of quality-assured ACT and to identify and report back on any potential stock-out issues.

### Targeting the ‘right’ outlets

The outlet survey showed that there were five types of non-governmental outlets that sold or distributed anti-malarial medicines in Eastern Myanmar, including oral artemisinin monotherapy. The census approached used by the ACTwatch study identified the relevancy of community health workers, private health workers, pharmacies, general retailers and mobile providers as anti-malarial-stocking outlets. The last three categories were recognized as being largely unregulated, meaning that these outlets did not receive any access to formal training or commodities from the government or other non-governmental organizations. This was also reflected in the outlet survey findings, which demonstrated relatively high availability of oral artemisinin monotherapy and low ACT availability among the anti-malarial stocking pharmacies, general retailers and mobile providers and high volumes of oral artemisinin monotherapy being distributed by these outlet types (and low-level of knowledge about recommended treatment for malaria). Thus, rather than reaching all outlet types, and given the urgency to implement rapid changes in the supply side across the Eastern areas, PSI product promoters targeted key outlet types: pharmacies, general retailers and mobile providers with the aforementioned provider supervision and regulation. To engage with mobile providers, details of their fixed location were obtained by the product promoters, and follow-up meetings were planned during the product promoter’s visit. As telephone coverage increased, the mobile providers were increasingly contacted by phone to secure follow-up visits.

### Nationwide sales

**Fig. 6:**
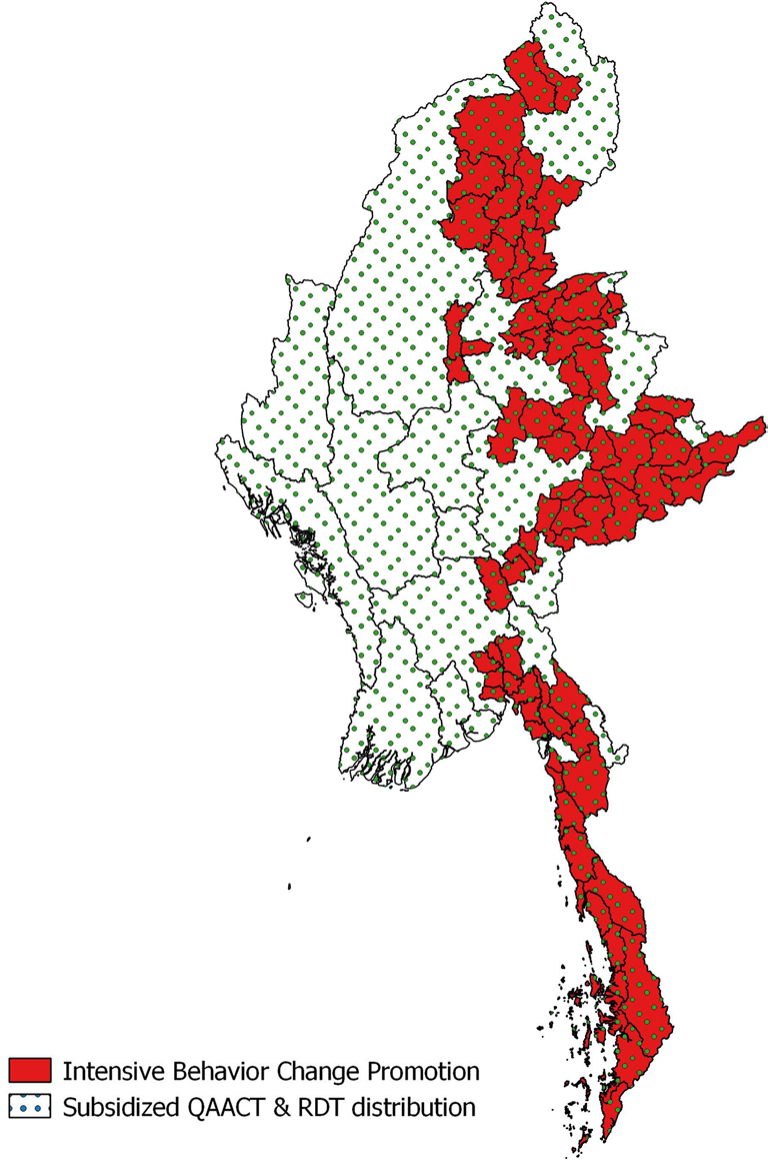
A map of the PSI target and sales area

While a targeted behavior change approach was implemented in Eastern Myanmar, sales of ACT were nationwide (Fig. 6). The rationale for this was both practical and epidemiological. Practically, containing private sector ACT sales within a particular region of Myanmar would have been extremely difficult; experience has shown that market forces draw the product where there is demand, and malaria is widespread in Myanmar, with 70 % of the population living in endemic areas. Epidemiologically, national sales of quality-assured ACT also made the most sense. Although there was no evidence of resistance outside of the eastern half of the country at the time of the implementation, it was recognized that continued availability and use of oral artemisinin monotherapy in western Myanmar could surely promote both the spread of, and potentially the *de novo* emergence of, artemisinin resistance. See Fig. 5, for an overview of the distribution system.

## Discussion

The AMTR project has been implemented since 2012, following supply chain study findings that showed high levels of oral artemisinin monotherapy sale and distribution in Myanmar. Since project inception, substantial improvements in the anti-malarial market landscape, have been observed and demonstrate the value of a high-level subsidy combined with supportive interventions. Recent findings from three survey rounds (2012, 2013 and 2014) illustrate the success of the project, with notable increases in quality-assured ACT availability and market share and declines in oral artemisinin monotherapy availability and distribution, mainly among priority outlets in the intervention area and, to a lesser extent, among priority outlets in the comparison area [14]. In addition to the outlet survey findings, there are several other lessons from the AMTR project that may be helpful for projects in other malaria-endemic settings struggling with private sector readiness and performance for appropriate malaria case management.

First, while the AMTR project included measuring change in supply side indicators over time, an important component was to influence and monitor the demand side, including patient fever treatment-seeking behavior, ACT treatment for suspected malaria and patient adherence to ACT treatment. However, due to low and declining rates of malaria prevalence in Myanmar, standard methods using population-based surveys to measure these indicators have not been useful in this context [15]. Despite screening several thousand households, sufficient numbers of patients with recent fever and patients who reportedly received anti-malarial treatment have been unobtainable through standard survey sampling methods. To address this issue, project indicators were revised, and demand-side evaluation methodologies were updated. The project now uses research methods to follow up with patients who received an ACT from priority and non-priority outlets to measure adherence.

Another challenge relates to drug policy for oral artemisinin monotherapy. Although banned by the FDA, there was no ban on oral artemisinin monotherapy sales or distribution and no mechanism for drug recall. Related to this, companies who were awarded 5-year distribution licenses just prior to the ban could legally continue to distribute for the duration of the license. While the AMTR project sought to remove all oral artemisinin monotherapy from the market, the lack of an enabling environment was a threat to the success of the intervention. Furthermore, the long shelf-life of oral artemisinin monotherapy means that this product can remain on the shelves for a long period. Conversely, the PSI-distributed quality-assured ACT has a shorter shelf life. Given the drop in Myanmar’s malaria caseload, providers may be incentivized to stock a malaria medicine with a longer shelf life and for which they can sell at a similar price to quality-assured ACT. In fact, recent outlet survey data from 2015 shows a rise in the availability of oral artemisinin monotherapy from 2014. Indeed, among priority outlets in the intervention area, availability of oral artemisinin monotherapy increased from 10 % in 2014 to 30 % in 2015. Similarly, market share of oral artemisinin monotherapy increased from around 21 % in 2014 to 26 % in 2015 [16]. This shows that, while substantial gains have been made over recent years, oral artemisinin monotherapy still stubbornly persists in Myanmar’s anti-malarial market, calling for the need for a complete ban on the importation, licensing, sale and distribution of oral artemisinin monotherapy as well as measures to enforce the ban.

Rapid diagnostic test (RDT) scale-up has only recently been introduced into the project and has been a challenging project component to implement. Changes in the political leadership and policies on the sale and distribution of malaria RDTs meant that, rather than using the private sector ACT supply chain for RDTs, RDTs had to be distributed free-of-charge in the private sector through product promoters. The AMTR project focused on launching a training program for private health providers to ensure correct RDT use, as well as mass media and interpersonal communication campaigns. The success of this arm of the intervention is yet to be evaluated, but the project’s need for flexibility and response to changing policies at the national level is important.

Finally, Myanmar’s anti-malarial market is constantly changing, making the application of appropriate market development strategies challenging and dynamic. Projecting commodities and supply chain management is a constant challenge. With declining malaria prevalence rates and increased coverage of malaria RDTs, the project strives to maintain a balance between the risk of drug expiry versus the risk of ACT stock-out. This is further complicated given challenges with estimating sales and outlet coverage between two distributors and geographical challenges in reaching certain areas of the country that are less accessible due to conflict and instability. There is a need to continually review available evidence and adjust strategies, inputs and targeting.

## Conclusions

Prior to the AMTR project intervention, in 2012, oral artemisinin monotherapy was available in over 50 % of anti-malarial-stocking outlets and accounted for 33 % of the market share. A rapid intervention was urgently required to address the availability and distribution of oral artemisinin monotherapy due to the threat to artemisinin drug efficacy. This required working with the private sector and primarily with unregulated outlet types in Myanmar in an attempt to change the most under-performing outlets, where oral artemisinin monotherapy accounted for almost half of the market share among those outlet types. Several lessons can be learned from the AMTR project that will be useful for other countries that are interested in redefining their anti-malarial market and engaging with the private sector.

## Abbreviations

AMTR: artemisinin monotherapy replacement malaria
BMGF: Bill and Melinda Gates Foundation
GMS: GMS Greater Mekong Subregion
IEC: information education and communication
MARC: Myanmar artemisinin resistance containment
PSI: Population Services International
DFID: UK Department For International Development
